# Identifying Longitudinal CD4:CD8 Ratio Trajectories Indicative of Chronic Renal Disease Risk among People Living with HIV: An Application of Growth Mixture Models

**DOI:** 10.3390/v15020385

**Published:** 2023-01-29

**Authors:** Alejandra Fonseca-Cuevas, Patrick Newsome, Lu Wang, Michelle Y. Chen, Chris G. Richardson, Mark Hull, Taylor McLinden, Silvia Guillemi, Rolando Barrios, Julio S. G. Montaner, Viviane D. Lima

**Affiliations:** 1 British Columbia Centre for Excellence in HIV/AIDS, Vancouver, BC V6Z 1Y6, Canada; 2 Department of Medicine, Faculty of Medicine, University of British Columbia, Vancouver, BC V5Z 1M9, Canada; 3 Department of Educational & Counselling Psychology & Special Education, University of British Columbia, Vancouver, BC V6T 1Z4, Canada; 4 Centre for Health Evaluation and Outcome Sciences, Providence Health Care, Vancouver, BC V6Z 1Y6, Canada; 5 Department of Family Medicine, University of British Columbia, Vancouver, BC V6T 1Z3, Canada

**Keywords:** CD4:CD8 ratio, HIV, chronic kidney disease, growth mixture modeling, decay model, risk factors

## Abstract

The incidence of chronic kidney disease (CKD) is increasing among people living with HIV (PLWH). Routine monitoring of indicators such as CD4:CD8 ratio might improve the early detection of CKD. Our objective was to identify clinically relevant CD4:CD8 ratio trajectories indicative of CKD risk. Participants were ≥ 18 years old, initiated antiretroviral therapy between 2000 and 2016, and were followed for ≥6 months until 31 March 2017 or last contact date. Outcome was incidence of CKD. Growth mixture models (GMMs) and decay models were used to compare CD4:CD8 ratio trajectories. Following GMM, 4547 (93.5%) participants were classified in Class 1 with 5.4% developing CKD, and 316 (6.5%) participants were classified in Class 2 with 20.9% developing CKD. The final model suggested that participants in Class 2 had 8.72 times the incidence rate of developing CKD than those in Class 1. Exponential decay models indicated a significant CD4:CD8 ratio decline among Class 2 participants who developed CKD. Among those who developed CKD in Class 2, starting at 5.5 years of follow-up, the slope of their ratio trajectory curve changed significantly, and the rate of decline increased dramatically. Routine monitored CD4:CD8 ratios can be an effective strategy to identify early CKD risk among PLWH.

## 1. Introduction

Since the introduction of antiretroviral therapy (ART), the life expectancy of people living with HIV (PLWH) has increased, which has led to higher incidence of age-associated conditions such as non-AIDS-related malignancies, liver, cardiovascular, cerebrovascular, and renal diseases in this population [1,2,3]. The complex process of aging, while living with HIV, presents many challenges for patients and healthcare providers, due to different psychosocial (e.g., social isolation), structural (e.g., access to healthcare resources, housing, food, and medications), and behavioral factors (e.g., substance use), and the cumulative toxicity of HIV/non-HIV medications increasing the complexity in the clinical management of individuals with multimorbidity [4]. Studies have shown that the management of individuals who present with a large number of medical issues can be quite complex, increasing the risk of polypharmacy and drug–drug interactions [5,6,7,8]. Since services and programs for PLWH with multimorbidity in different settings can be highly disconnected, they are inefficient in adequately achieving those who need care, adding to the complexity in the clinical management of these individuals. One of the most effective strategies to improve the overall health of patients is to change the way clinicians and other healthcare providers deliver care to a population. Care should pivot from focusing on individual conditions to a holistic approach that considers multiple interrelated health conditions and health determinants that contribute to an excessive disease burden [6,9,10]. Therefore, due to the complexity in treating these patients, there is an urgent need to organize care by integrating multiple healthcare practitioners and services, which allows patients to receive the care they need, when they need it, in a cost-effective way to reduce morbidity and premature mortality in this population [11,12,13].

One of the most commonly diagnosed age-associated conditions among PLWH is chronic kidney disease (CKD), being three times more likely to develop among PLWH than the general population [14,15,16,17]. In British Columbia (BC), CKD affects 13.5% of PLWH compared to 1.9% of HIV-negative individuals [17]. Risk factors for CKD include low CD4 cell counts, high HIV viral loads, and common comorbidities such as hypertension and type II diabetes mellitus [18,19,20,21,22,23,24]. Some antiretrovirals may increase the risk of CKD due to their nephrotoxicity (mostly older drugs such as tenofovir disoproxil fumarate, lopinavir/atazanavir/darunavir boosted with lopinavir, and to a lesser extent tenofovir alafenamide) and others are known to inhibit the excretion of serum creatinine by proximal renal tubular cells by the blockade of specific transporters, which are not toxic to the cell but are linked to changes in serum creatinine levels (e.g., dolutegravir, rilpivirine, cobicistat) [23,25,26]. Since CKD may lead to end-stage renal disease and death, therefore early identification of CKD is necessary to prevent disease progression [14].

Current guidelines for CKD screening rely on annual/biannual urine and blood tests to obtain glomerular filtration rates (eGFR) and albumin/creatinine ratios (ACR) [24,27,28,29,30,31]. However, eGFR and ACR estimations depend on serum creatinine from muscle which is influenced by muscle mass, nutritional status, age, race, the amount resorbed in the kidney, and by some ART drugs leading to possible limitations on the early recognition of CKD among PLWH [27,28,32,33]. Other routinely monitored tests among PLWH are markers of chronic inflammation and immune senescence, and they may aid in identifying early CKD, such as the CD4:CD8 ratio [29,34,35,36,37,38]. Studies have shown that CD4:CD8 ratios are linked to chronic infection resulting in persistent immune system activation which predisposes people to a higher risk of chronic comorbidities [39]. Persistent CD4:CD8 ratios less than 0.3 have been shown to be independently associated with increased risk of non-AIDS-related events and mortality [1,37,40]. Although these ratios provide good prognostic information on non-AIDS related events, limited research has been conducted to assess whether CD4:CD8 ratio are linked to any disease-specific burden [1,40,41,42,43,44,45].

Traditional methods have used CD4:CD8 ratios as a prognostic factor for disease progression and inflammation using different predetermined cut-off points [40,46,47,48,49,50]. However, the use of different cut-offs for CD4:CD8 ratio classification may lead to inconsistencies, thus compromising generalizability and comparability of results across studies [51]. Identifying CD4:CD8 ratio trajectories specifically linked to CKD patients could improve early recognition of CKD among PLWH, and support the implementation of interventions and strategies to closely monitor PLWH at higher risk of CKD [52]. Thus, the objective of this study was to identify clinically relevant CD4:CD8 ratio trajectories indicative of early CKD risk among PLWH. We also explored the effect of sex on the risk of CKD.

## 2. Materials and Methods

### 2.1. Study Setting and Data

Data were obtained from the BC Seek and Treat for Optimal Prevention of HIV/AIDS (STOP HIV/AIDS) population-based cohort with access to data on PLWH since their earliest date indicating HIV seropositivity [53]. This cohort is based on a longitudinal data linkage of several provincial databases including the BC Center for Excellence in HIV/AIDS Drug Treatment registry, the laboratory database, and several provincial administrative health databases as explained in the Supplementary Materials [53,54,55,56,57,58,59,60,61,62]. Data captured in the STOP HIV/AIDS linkages include sociodemographic factors such as sex, age, and geographic location, ART treatment information, healthcare use, and diagnostic and laboratory biomarkers such as CD4 cell counts, CD4:CD8 ratio, and HIV viral load. The last data update had data until 31 March 2017.

### 2.2. Study Design

A population-based cohort study design with the following inclusion criteria: (i) age ≥18 years, (ii) ART initiation between 1 January 2000 and 30 September 2016, (iii) no previous supervised ART interruptions, (iv) no participation in a blinded ART-related clinical trial, and (v) no previous diagnosis of CKD. Additionally, in order to fit the models, we required (i) at least six months of follow-up, (ii) available HIV viral load and CD4 measurements during the follow-up, and (iii) at least two CD4:CD8 ratio measurements throughout the study. Eligible participants were followed until 31 March 2017, the last contact date (i.e., the last filled ART prescription refill date, the last available laboratory test date or the date of last encounter with the healthcare system), or the date of death (all-causes).

### 2.3. Outcome and Study Covariates

The outcome was incidence of CKD identified using a case finding algorithm published by the BC Ministry of Health based on physician claims and hospitalizations with a diagnostic code for CKD Supplementary Materials, Table S1 [60]. To estimate incidence, we applied a washout period of five years before first ART date. For participants lacking five years of information prior to their first ART date, a washout period of five years after their earliest encounter with the healthcare system was used. CD4:CD8 ratios were measured every six months.

Time-invariant covariates measured at baseline included: sex (female, male), age (continuous), ART naïve (yes, no), and a categorical indicator reflecting a cohort effect (years 2000–2004, 2005–2010, 2011–2016). Time-invariant covariates measured at the end of follow-up included: history of substance use disorder (SUD) (yes, no), duration of hypertension, diabetes mellitus, and cardiovascular disease (CVD) (no disease, < median duration, ≥ median duration), area under the HIV viral load curve [63], proportion of time on protease inhibitors (PI), proportion of time on non-nucleoside reverse transcriptase inhibitors (NNRTI), proportion of time on tenofovir disoproxil fumarate (TDF), and follow-up time (continuous). CD4 nadir (continuous) was measured one year before the end of follow-up. Case-finding algorithms used to identify participants with SUD, hypertension, diabetes mellitus and CVD are described in the Supplementary Materials Table S1 Please note that, beside TDF, we did not investigate other specific antiretrovirals known to increase the risk of CKD [20,21,22]. The variables included in this study were chosen a priori based on the clinical literature and treatment guidelines describing factors known to influence CKD risk.

### 2.4. Statistical Analyses

We conducted bivariable analyses between risk factors and CKD using Pearson’s chi-square or Fisher’s exact test for categorical variables (depending on cell count), and Wilcoxon’s Rank Sum test for continuous variables. This study consisted of a two-pronged analysis:

#### 2.4.1. Growth Mixture Modeling

To investigate the patterns of CD4:CD8 ratio trajectories among participants with and without CKD, we applied growth mixture modeling (GMM) [52,64,65]. This type of modeling allows the identification of unobserved subpopulations or classes based on distinctive trajectories of CD4:CD8 ratios. First, we fit unconditional models with a different number of latent classes, and with linear (in case the ratio changes linearly over time) and quadratic (in case there is a non-linear pattern over time) growth factors; these terms were introduced one at a time. Second, we focused on examining the relationships between covariates and latent classes by fitting the GMM including covariates. Adding covariates to the model helps us understand the key characteristics of each group and how different covariates may affect the longitudinal trajectory within each class. To examine the fitness of the models, we used the Akaike information criteria (AIC), Bayesian information criteria (BIC), sample size-adjusted BIC (SBIC), entropy (range 0 to 1), adjusted Lo–Mendell–Rubin likelihood ratio test (LMR-LRT), and class size. A LMR-LRT with *p*-value of <0.05 was deemed significant while lower values of AIC, BIC and SBIC indicated better fit [66,67]. Third, we compared the incidence of CKD using two versions of the selected model, one following an unconditional approach (without covariates) and the other including covariates. To analyze the association between the outcome (CKD incidence) and the latent classes, we assigned participants to their most likely classes and then included this variable as an explanatory variable in the Poisson regression to model the incidence CKD rate per 1000 person-years between the distinct trajectories. Last, to understand the key characteristics of participants in each class and how different covariates may affect the CD4:CD8 ratio trajectories, we conducted bivariable analyses between covariates and the latent classes stratified by CKD status. Analyses were conducted with SAS version 9.4 (SAS Institute Inc., Cary, NC, USA) and Mplus v.8.1 (Los Angeles, CA, USA).

#### 2.4.2. Decay Model

Based on the results from the GMM, we estimated CD4:CD8 ratio trajectories to assess whether there are early indications of participants being at a high risk of CKD and potential cut-off time points of intervention. CD4:CD8 ratio measurements were obtained longitudinally from study baseline until the end of follow-up. We modeled and compared the CD4:CD8 ratio depletion trajectories among the identified latent classes and CKD status. The natural logarithm of CD4:CD8 ratios were modeled using a non-linear mixed effects model [68,69]. The follow-up period was modeled using exponential decay function, assuming a Gaussian distribution (identity link), an AR(1) correlation structure, and a random intercept. The exponential decay model is described as follows:

$$ln{\left(CD4:CD8\right)}_{i,t}\sim \;\gamma e^{-R*t_{i}}+C_{0}+b_{0i}+\varepsilon_{i,t}$$

where *t* represents each of the six-month intervals, *i* represents each participant at the study, $\varepsilon_{i}$ is the random error distributed as *Normal* (0, *D_i_*), where *D* is the covariance matrix and $b_{0i}\;$as *N* (0, 𝜖^2^). The exponential decay function was modeled through $\gamma e^{-R*t}$, where γ and *R* are coefficients of this function. Loss rate was modeled using the following equations.

$$y=ln\left(r\right)$$

$$\frac{dy}{dt}=\frac{d\;ln\left(r\right)}{dt}$$

$$\frac{dy}{dt}=\frac{1}{r}\times \frac{dr}{dt}$$

where r is the CD4:CD8 ratio, $\frac{dr}{dt}$ is the CD4:CD8 rate and $\frac{dy}{dt}$ is the $ln\left(CD4:CD8\right)$ rate. These analyses were conducted in R© version 4.0.5.

## 3. Results

The STOP HIV/AIDS cohort includes 15,599 participants, of which 8286 were excluded since they were not eligible for the study. Thus, out of the remaining 7313 patients, 2450 were eliminated since they did not fulfill the data requirements for the modeling. Therefore, 4863 participants were included in our study Supplementary Materials, Figure S1. Those excluded (i.e., 2450) were more likely to be female, with a history of SUD, not naïve to ART, enrolled in the cohort between 2011 and 2016 and of an older age Supplementary Materials, Table S2. Most of the included participants were male (82.2%), ART naïve (89.6%), without a history of SUD (57.3%), hypertension (87.2%), diabetes mellitus (92.0%), and CVD (93.1%). The median follow-up time was 6.45 (25th, 75th percentiles: 3.57, 9.89) years, and the median number of CD4:CD8 measurements was 8 (25th, 75th percentiles: 4, 15). A total of 310 (6.4%) participants developed CKD throughout the study. Bivariable analyses revealed that participants who developed CKD and those who did not differ across all measured characteristics, except age at the end of follow-up, and the proportion of time on NNRTI Table 1. Participants who developed CKD were more likely to be female, naïve to ART, enrolled in the cohort between 2000 and 2004, with a history of SUD, hypertension, diabetes and CVD, older at study entry, higher baseline HIV viral load, shorter exposure to TDF, and longer exposure to PI.

### 3.1. CD4:CD8 Ratio Trajectories and CKD Status

We built different GMMs to model the CD4:CD8 ratio measurements over time. Based on the goodness-of-fit statistics found in the Supplementary Materials Table S3, the final model included two classes (linear) with time-invariant covariates Supplementary Materials Figure S2. Having two classes means that there are two groups in our population, and they differ in the CD4:CD8 ratio trajectory in a “particular way”, which we try to understand based on the following analyses. We also compared the rate of renal disease of the two-class (linear) model with and without time-invariant covariates Table 2. Our results showed that in both models, participants in Class 2 had a higher rate of CKD than those in Class 1. For the final model, i.e., two-class (linear) model with time-invariant, those assigned Class 2 had 8.72 times the rate (95% CI 6.64–11.44) of developing incident CKD than those in Class 1.

Based on the final model, we plotted the CD4:CD8 ratio trajectories for each class Supplementary Materials Figure S3 and performed bivariable analyses Table 3. A total of 4547 (93.5%) participants were classified in Class 1 with 244 (5.4%) of them developing incident CKD, and 316 (6.5%) were classified in Class 2 with 66 (20.9%) developing incident CKD. Our bivariable analyses suggested that among Class 2 participants, those who develop CKD were more likely to have history of SUD, be ART naïve, enrolled in the cohort between 2000 and 2004, younger at baseline, with a lower CD4 nadir closer to the end of follow-up, and a shorter time on TDF (*p*-values <0.05).

### 3.2. CD4:CD8 Ratio Decay among Classes and CKD Status

**Figure 1:**
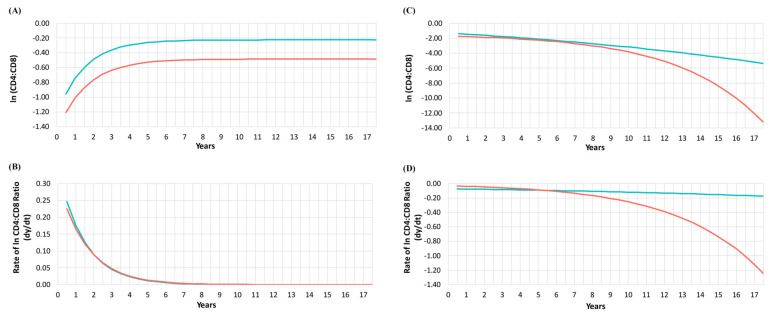
$ln\left(CD4:CD8\right)\;$trajectory and loss rate plots per six months by chronic kidney disease (CKD) status. (**A**) Estimated trajectory in Class 1. (**B**) Estimated trajectory in Class 2. (**C**) Estimated loss rate for Class 1. (**D**) Estimated loss rate for Class 2. Red line CKD and Green line no CKD.

We fit an exponential decay function model to estimate the CD4:CD8 ratio trajectories by GMM class and CKD status. A total of 60,869 CD4:CD8 measurements were included with 59,191 belonging to Class 1 and 1678 to Class 2. The models and goodness-of-fit assessments can be found in the Supplementary Materials Table S3, Figure S4. Figure 1a,b present the $ln\left(CD4:CD8\right)$ trajectory, and Figure 1c,d present the $ln\left(CD4:CD8\right)\;$loss rate over time, for every 6-month period. We observed that the estimated trajectory of $ln\left(CD4:CD8\right)$ ratio of participants who developed CKD differed significantly between Classes 1 and 2. Those in Class 1 experienced a rapid increase and after 7.5 years, the ratio reached a plateau Figure 1a. Please note that although the rate of increase between those with and without CKD was very similar, those with CKD in Class 1 had significantly lower ratios. For those in Class 2, we observed a decline in $ln\left(CD4:CD8\right)$ among those with and without CKD. However, among those who developed CKD, starting around 5.5–6.5 years of follow-up Figure 1b,d, the slope of their ratio trajectory curve changed significantly, and the rate of decline increased dramatically. Data for Figure 1 can be found in Tables S4 and S5, as well as the original values for the ratio in this analysis.

### 3.3. Sex-Based Analysis

**Figure 2:**
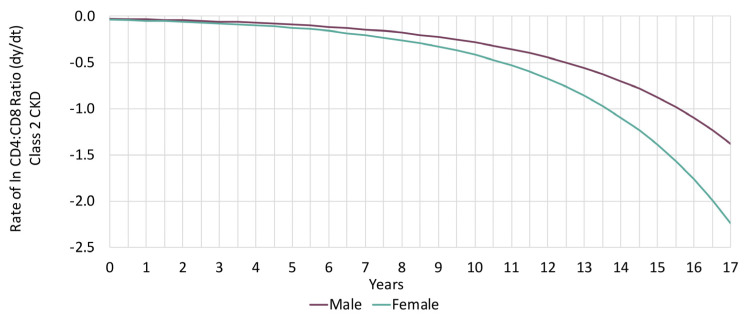
$ln\left(CD4:CD8\right)\;$trajectory plot per six months among participants who developed chronic kidney disease (CKD) in Class 2 by sex.

We conducted a sensitivity analysis by sex at birth. Please note that to run GMM or any other statistical model, we need power and since we have very few females in our study, we could only run descriptive analyses and fit the decay model for Class 2 only including sex as a covariate. We noticed that the rate of CKD is 53% higher in females (Table 4) and the rate of decline in $ln\left(CD4:CD8\right)$ among Class 2 with CKD was also more accelerated among females (Figure 2).

## 4. Discussion

Using routinely monitored tests such as the CD4:CD8 ratio, this study identified clinically relevant subpopulations of PLWH who are at increased risk of incident CKD. Our novel use of GMM allowed us identify higher CKD rates among participants classified into the Class 2 trajectories. Furthermore, our subsequent decay functions and CD4:CD8 ratio loss rates also suggested that, among Class 2 participants, there was a significant CD4:CD8 ratio decline among those who developed CKD starting at 5.5–6.5 years of follow-up (a CD4:CD8 ratio < 0.10); or a decline of 45% from baseline values. This finding is interesting and puzzling. The usual pattern for patients is to improve their CD4:CD8 ratio once they start ART [70]. For those with advanced HIV prior to the start of ART (e.g., low CD4 nadir, prior opportunistic infection), the improvement usually seen in the CD4:CD8 ratio is minor [2,70]. In this study, CKD incidence was higher among participants with the poorest ratio response in both classes, especially those in Class 2. We also observed that in our study, females had a higher CKD incidence, and the rate of decline in CD4:CD8 was more accelerated than in males. Some studies have shown that females, especially done of child-bearing age may experience higher rates of CKD due to biological factors as well as inequities in accessing care for early diagnosis of kidney disease [71,72]. One question that comes to mind is whether HIV-related factors drove the poor response seen in the CD4:CD8 ratio. In our study, the AUCVL in all groups did not indicate that poor virologic control influenced our results. Additionally, the effect may have been driven by a loss of memory CD4 from living with HIV for a longer time, as indicated in our results. Third, we controlled for the effect of CD4 nadir and antiretrovirals known to be risk factors for CKD, and the our CD4:CD8 GMM model was still able to distinguish those more likely to develop CKD. Last, suppose that non-HIV-related factors are involved in the observed trends in the CD4:CD8 ratio, especially in Class 2. In this case, the question that remains is, as CKD progresses, is there a biological mechanism that predicts immunologic non-response among PLWH?

The use of eGFR and ACR has been identified as gold standard to identify and monitor CKD [73]. However, these measurements can yield low sensitivity in detecting early CKD, especially among PLWH, participants with certain comorbidities (e.g., chronic liver disease, obesity) [30,74,75], and external factors such as muscle mass, nutritional status, age, and race. Among PLWH, routinely monitored CD4:CD8 ratio measurements have been identified as emerging biomarkers due to its low values being associated with increased risk of morbidity and mortality [39,50,76,77,78]. Previous studies have focused on using the CD4:CD8 ratio as a marker of greater risk of cardiovascular events, non-AIDS defining cancers, diabetes, and hypertriglyceridemia [37,40,42,46]. These studies have also used predetermined CD4:CD8 ratio cut-offs to assess the risk of morbidity and mortality [79]. However, given our PLWH population and their variability in demographic, clinical, and behavioral characteristics, it is key to use an approach that accounts for the heterogeneity of participants, such as GMM [52]. Our GMM allowed us to examine the dynamic changes in CD4:CD8 ratio during treatment and identify the diversity in trajectories while accounting for traditional risk factors such as sex, history of SUD, ART regimen and presence of comorbidities. This model aided in the identification of clinically relevant subpopulations of PLWH with higher CKD risk [46], through the different trajectories in CD4:CD8 ratio. Please note that one of the factors that we explored in this study was time exposed to different classes of ART and TDF. As expected, longer time exposed to PIs was associated with a higher risk of CKD. Please note that, during the study, a large proportion of PLWH received ritonavir-boosted atazanavir followed by ritonavir-boosted lopinavir. We also found that those who developed CKD received TDF for a shorter time. This finding is related to confounding by indication, i.e., for some participants, TDF is rapidly discontinued likely due to abnormal renal test results.

Our study has some important strengths. First, the use of linked datasets comprised of different administrative health databases allowed us to obtain clinical information necessary to account for population characteristics, using GMM, and estimate CD4:CD8 ratio trajectories. Second, we used a relatively long washout period (i.e., five years) to estimate CKD incidence and minimize the risk of capturing prevalent cases. Third, we were able to evaluate cumulative exposure to ART known to increase the risk of CKD. However, there are also some limitations to consider. First, our estimates relied on at least two measurements of CD4:CD8 ratio per participant. Thus, participants with only one measurement were omitted from the study. Second, we were unable to adjust for covariates in our CD4:CD8 ratio decay model due to sample size limitations. However, we conducted goodness-of-fit assessments to choose our models, and the model showed good performance with the standardized residuals and the fitted values being correlated with the observed values. Third, we used AUCVL to summarize the overall viral load trajectories of each participant in our study. The measure as calculated may be subjected to selection bias since participants have different measures of viral load over time [80]. However, we showed in the table comparing the characteristics of those belonging to each of the classes, that the AUCVL was not statistically significantly different between the comparison groups, and therefore, we believe that this bias did not play a significant role in our results. Last, although our models yield good estimates, we did not have access to other factors that may explain the risk of CKD (e.g., race/ethnicity, hepatitis C co-infection, smoking) in our population [30].

## 5. Conclusions

This study demonstrated the potential use of the CD4:CD8 ratio as a complementary measure to eGFR and ACR to aid the early identification of PLWH who may be at elevated risk of developing CKD, and could therefore benefit from closer longitudinal monitoring of their renal function.

## Figures and Tables

**Table 1:** Study population characteristics of the final analytical sample of eligible participants (*n* = 4863), stratified by chronic kidney disease status.

	Overall N = 4863	Did Not Develop CKD N = 4553	Developed CKD N = 310	
Risk factors	N (%)	N (row %)	N (row %)	*p*-value
Sex				0.0010
Female	867 (17.8)	789 (91.0)	78 (9.0)	
Male	3996 (82.2)	3764 (94.2)	232 (5.8)	
SUD				<0.0001
No	2784 (57.3)	2674 (96.0)	110 (4.0)	
Yes	1685 (34.7)	1495 (88.7)	190 (11.3)	
Unknown	394 (8.1)	384 (97.5)	10 (2.5)	
ART naïve				0.0009
Yes	4359 (89.6)	4064 (93.2)	295 (6.8)	
No	504 (10.4)	489 (97.0)	15 (3.0)	
Year of baseline date				<0.0001
2000–2004	1026 (21.1)	876 (85.4)	150 (14.6)	
2005–2010	2012 (41.4)	1884 (93.6)	128 (6.4)	
2011–2016	1825 (37.5)	1793 (98.2)	32 (1.8)	
Hypertension during follow-up				<0.0001
No	4241 (87.2)	3996 (94.2)	245 (5.8)	
Yes	622 (12.8)	557 (89.5)	65 (10.5)	
Diabetes during follow-up				<0.0001
No	4476 (92.0)	4209 (94.0)	267 (6.0)	
Yes	387 (8.0)	344 (88.9)	43 (11.1)	
CVD during follow-up				<0.0001
No	4528 (93.1)	4271 (94.3)	257 (5.7)	
Yes	335 (6.9)	282 (84.2)	53 (15.8)	
Hypertension duration				<0.0001
No disease	4241 (87.2)	3996 (94.2)	245 (5.8)	
<median duration (4.8 years)	311 (6.4)	271 (87.1)	40 (12.9)	
≥median duration (4.8 years)	311 (6.4)	286 (92.0)	25 (8.0)	
Diabetes duration				<0.0001
No disease	4476 (92.0)	4209 (94.0)	267 (6.0)	
<median duration (4.2 years)	193 (4.0)	170 (88.1)	23 (11.9)	
≥median duration (4.2 years)	194 (4.0)	174 (89.7)	20 (10.3)	
CVD duration				<0.0001
No disease	4528 (93.1)	4271 (94.3)	257 (5.7)	
<median duration (3.2 years)	167 (3.4)	133 (79.6)	34 (20.4)	
≥median duration (3.2 years)	168 (3.5)	149 (88.7)	19 (11.3)	
	Median (Q1, Q3)	Median (Q1, Q3)	Median (Q1, Q3)	*p*-value
Age at baseline (year)	41 (34, 49)	41 (34, 49)	43 (36, 52)	0.0010
Age at the end of follow-up (year)	49 (41, 56)	49 (41, 56)	49 (41, 57)	0.6241
CD4 Nadir (cells/µL)	250 (110, 410)	260 (120, 420)	110 (40, 200)	<0.0001
CD4 Nadir within one year prior to the end (cells/µL)	480 (300, 670)	500 (320, 680)	210 (80, 387)	<0.0001
Baseline viral load (log_10_ copies/mL)	4.7 (3.7, 5.0)	4.6 (3.6, 5.0)	5.0 (4.4, 5.0)	<0.0001
AUVLC (log_10_ copy-years/mL)	12.5(6.9, 19.5)	12.6 (7.0, 19.6)	11.9 (5.6, 17.5)	0.0054
Follow-up time (year)	6.4 (3.6, 9.9)	6.6 (3.7, 10.0)	4.5 (2.4, 8.1)	<0.0001
Proportion of time on TDF	0.7 (0.1, 0.9)	0.7 (0.2, 1.0)	0.3 (0, 0.8)	<0.0001
Proportion of time on PI	0.2 (0, 0.8)	0.2 (0, 0.8)	0.5 (0, 0.9)	<0.0001
Proportion of time on NNRTI	0.0 (0, 0.8)	0.0 (0, 0.8)	0.0 (0, 0.6)	0.2729

Note: We present row percentages for easier interpretation of factors associated with those who developed or not chronic kidney disease, Q1, Q3: 25th and 75th percentiles, CKD: chronic kidney disease, SUD: history of substance use disorder, ART: antiretroviral therapy, CVD: cardiovascular disease, AUVLC: area under the plasma viral load curve, TDF: tenofovir disoproxil fumarate, PI: protease inhibitors, NNRTI: non-nucleoside reverse transcriptase inhibitors.

**Table 2:** Comparison of chronic kidney disease rates of participants assigned Class 1 and 2 using the two-class linear model with and without time-invariant covariates.

	Number of Participants	Number of Participants Who Did Not Developed CKD	Number of Participants Who Developed CKD	CKD Rate, 95% CI (per 1000 Person-Years)	Rate Ratio (95% CI)
Two-class linear model (unconditional)					
Class 1	4725	4442	283	8.49 (7.55–9.53)	Reference
Class 2	138	111	27	32.70 (22.43–47.69)	3.85 (2.60–5.72)
Two-class linear model (time-invariant covariates)					
Class 1	4547	4303	244	7.36 (6.49–8.34)	Reference
Class 2	316	250	66	64.16 (50.41–81.67)	8.72 (6.64–11.44)

Note: 95% CI: 95% confidence interval, CKD: chronic kidney disease.

**Table 3:** Descriptive statistics of the study population by CD4:CD8 class identified by two-class linear model with time-invariant covariates on chronic kidney disease (CKD) status.

	Class 1			Class 2		
	CKD No (*n* = 4303)	CKD Yes (*n* = 244)		CKD No (*n* = 250)	CKD Yes (*n* = 66)	
Risk factors	N (row %)	N (row %)	*p*-Value	N (row %)	N (row %)	*p*-value
Sex			0.0090			0.2790
Female	726 (92.7)	57 (7.3)		63 (75.0)	21 (25.0)	
Male	3577 (95.0)	187 (5.0)		187 (80.6)	45 (19.4)	
SUD			<0.0001			0.0084
No	2567 (96.5)	94 (3.5)		107 (87.0)	16 (13.0)	
Yes	1362 (90.6)	141 (9.4)		133 (73.1)	46–49 *	
Unknown	374 (97.7)	9 (2.3)		10 (90.9)	<5 **	
ARV naïve			0.0138			0.0129
Yes	3843 (94.4)	230 (5.6)		221 (77.3)	62–64 *	
No	460 (97.0)	14 (3.0)		29 (96.7)	<5 **	
Year of baseline date			<0.0001			<0.0001
2000–2004	808 (87.5)	115 (12.5)		68 (66.0)	35 (34.0)	
2005–2010	1814 (94.5)	106 (5.5)		70 (76.1)	22 (23.9)	
2011–2016	1681 (98.7)	23 (1.3)		112 (92.6)	9 (7.4)	
Hypertension duration			<0.0001			0.9990
No disease	3764 (95.3)	184 (4.7)		232 (79.2)	61 (20.8)	
<median duration (4.8 years)	256 (87.7)	36 (12.3)		15 (78.9)	<5 **	
≥median duration (4.8 years)	283 (92.2)	24 (7.8)		<5 (75.0)	<5 **	
Diabetes duration			<0.0001			0.3850
No disease	3970 (95.1)	205 (4.9)		239 (79.4)	62 (20.6)	
<median duration (4.2 years)	160 (88.9)	20 (11.1)		10 (76.9)	<5 **	
≥median duration (4.2 years)	173 (90.1)	19 (9.9)		1 (50.0)	<5 **	
CVD duration			<0.0001			0.5507
No disease	4031 (95.4)	195 (4.6)		240 (79.5)	62 (20.5)	
<median duration (3.2 years)	126 (80.3)	31 (19.7)		6–9	<5 **	
≥median duration (3.2 years)	146 (89.0)	18 (11.0)		<5 **	<5 **	
	Median (Q1, Q3)	Median (Q1, Q3)	*p*-Value	Median (Q1, Q3)	Median (Q1, Q3)	*p*-value
Age at baseline	41 (34–49)	44 (37–53)	<0.0001	43 (34–49)	38 (31–45)	0.0048
CD4 Nadir within one year prior to the end (cells/µL)	520 (360–690)	270 (150–440)	<0.0001	70 (20–130)	30 (10–60)	<0.0001
AUVLC (log_10_ copy-years/mL)	12.7 (7.4–19.8)	12.2 (6.0–17.9)	0.0587	8.9 (3.6–16.0)	8.7 (3.8–15.4)	0.9469
Follow-up time (year)	6.9 (4.0–10.2)	5.8 (2.9–8.5)	<0.0001	2.5 (1.4–4.9)	2.5 (1.1–3.9)	0.3473
Proportion of time on TDF	0.7 (0.2–1.0)	0.4 (0–0.9)	<0.0001	0.2 (0–0.6)	0.2 (0–0.4)	0.0286
Proportion of time on PI	0.2 (0–0.8)	0.6 (0–0.9)	<0.0001	0.2 (0–0.5)	0.2 (0–0.5)	0.6681
Proportion of time on NNRTI	0.1 (0–0.8)	0.04 (0–0.8)	0.5783	0 (0–0.2)	0 (0–0.2)	0.1088

Note: We present row percentages for easier interpretation of factors associated with those who have or not chronic kidney disease, Q1, Q3: 25th and 75th percentiles, CKD: chronic kidney disease, SUD: history of substance use disorder, ART: antiretroviral therapy, CVD: cardiovascular disease, AUVLC: Area under the plasma viral load curve, TDF: tenofovir disoproxil fumarate, PI: protease inhibitors, NNRTI: non-nucleoside reverse transcriptase inhibitors. ^*^ Due to data privacy, we provided a range due the cells with <5 participants, ** Due to data privacy, we replaced the exact numbers if the cell contained <5 participants.

**Table 4:** Comparison of chronic kidney disease (CKD) rates of by sex.

Sex	Number of Participants	Number of Participants Who Developed CKS	CKD Rate, 95% CI (per 1000 Person-Years)	Rate Ratio (95% CI)
Male	3996	232	8.28 (7.28–9.41)	Ref
Female	867	78	12.68 (10.16–15.83)	1.53 (1.19–1.98)

Note: 95% CI: 95% confidence interval, CKD: chronic kidney disease.

## Data Availability

The British Columbia Center for Excellence in HIV/AIDS is prohibited from making individual-level data available publicly due to provisions in our service contracts, institutional policy, and ethical requirements. The British Columbia Center for Excellence in HIV/AIDS data are not available externally due to prohibitions in service contracts with our funders or data providers. Institutional policies stipulate that all external data requests require collaboration with a BC Center for Excellence in HIV/AIDS. For more information regarding data access, please contact Mr. Mark Helberg, the Senior Director, Internal and External Relations & Strategic Development of the British Columbia Center for Excellence in HIV/AIDS, at mhelberg@bccfe.ca. In his position, he oversees some of the daily operations from the Center and any data access request that may arise.

## Supplementary Materials

The following supporting information can be downloaded at: https://www.mdpi.com/article/10.3390/v15020385/s1.
