# Supplementary material for: Identifying Longitudinal CD4:CD8 Ratio Trajectories Indicative of Chronic Renal Disease Risk among People Living with HIV: An Application of Growth Mixture Models
Source: Viruses. 2023 Jan 29;15(2):385. doi: 10.3390/v15020385 (PMC9963117; doi:10.3390/v15020385)
Supplement: Supplementary file 1 [file viruses-15-00385-s001.zip › viruses-2119069-supplementary.pdf]

Identifying Longitudinal CD4:CD8 Ratio Trajectories Indicative of Chronic Renal Disease Risk among People Living with HIV: An Application of Growth Mixture Models

Table of Contents

STOP Seek and Treat for Optimal Prevention of HIV/AIDS (STOP HIV/AIDS) Information..... 2

Supplementary Material Table S1. Comorbidities analyzed and their case-finding algorithms 4

Supplementary Material Figure S1. Flowchart describing study participant selection from the STOP HIV/AIDS PLWH cohort.. 12

Supplementary Material Table S2. Descriptive statistics of excluded and included participants. 13

Supplementary Material Figure S2. Diagram of growth mixture model estimating growth parameters (i.e., intercept, slope) for linear trajectory shapes. 15

Supplementary Material Figure S3. Plots of the two-class (linear) models from the final Growth Mixture Models. A) Class 1 (N= 4547), B) Class 2 (N= 316). 16

Decay model 17

Supplementary Material Table S3. Goodness-of-fit parameters for the exponential decay model without covariates. 17

Supplementary Material Figure S4. CD4:CD8 Decay Model and Goodness-of-fit A) Class 1, no chronic kidney disease, B) Class 1, with chronic kidney disease, C) Class 2, no chronic kidney disease, D) Class 2, with chronic kidney disease.. 18

Supplementary Material Table S4. $lnCD4:CD8$ trajectory and loss rate plots per six months by chronic kidney disease status among Class 2 participants.. 19

Supplementary Material Table S5. $lnCD4:CD8$ trajectory and loss rate plots per six months by chronic kidney disease status among Class 1 participants.. 21

Supplementary Material Table S6. $lnCD4:CD8$ trajectory and loss rate plots per six months by chronic kidney disease status among Class 2 participants by sex. 23

References cited in the Supplemental Material 26

STOP Seek and Treat for Optimal Prevention of HIV/AIDS (STOP HIV/AIDS) Information

Data for STOP HIV/AIDS population-based cohort was derived from various linkages between provincial administrative databases.

Data Steward: British Columbia Centre for Disease Control [1,2]

i. Provincial HIV/AIDS Surveillance Database: surveillance database that collates all HIV laboratory testing, new HIV diagnosis and occurrence of AIDS-defining illnesses data;

Data Steward: British Columbia Centre for Excellence in HIV/AIDS [3,4]

ii. Drug Treatment and Laboratory Databases: database that collates all antiretroviral dispensing data, plasma viral load testing, drug resistance testing, occurrence of AIDS-defining illnesses, ~85% of CD4 cell count measurements, and patient demographic information;

Data Steward: British Columbia Ministry of Health [5]

iii. The Medical Services Plan (MSP) billing database: database that captures HIV and non-HIV-related inpatient and outpatient services provided by physicians and supplementary health care practitioners, as well as diagnostic procedures. MSP also contains cost associated with claims paid through fee-for-service and the Alternative Payment Program;

iv. Home and Community Care database: database that captures a variety of services including hospice and home nursing care, adult day services, assisted living, respite care, residential and convalescent care

v. Mental Health Services database: database that captures utilization of mental health services including fee-for-service, institutional care, community clinics and acute care.

vi. Addictions Information Management Systems: database that captures referral to treatment for alcohol, drug or gambling addictions.

vii. The PharmaNet database: a real-time system that captures all prescriptions for drugs and medical supplies dispensed from community pharmacies in BC and prescriptions dispensed from hospital outpatient pharmacies use at home. Note that this database does not capture antiretroviral dispensing data;

viii. The Client Roster or Consolidation File: database that captures individual demographic and geographic data and is used to construct population denominators.

Data Steward: Canadian Institute for Health Information [6]

The Discharge Abstract Database (DAD): database that captures all discharges, transfers and deaths of in-patients and day surgery patients from acute care hospitals across BC;

Data Steward: British Columbia Vital Statistics Agency [7]

ix. The Vital Statistics database: records death information of all BC's residents.

Supplementary Material Table S1. Comorbidities analyzed and their case-finding algorithms

Chronic Age-related Comorbidities		Case Definitions	Diagnostic, Procedure, and/or Drug Codes	References
Cardiovascular Disease (CVD)	Acute myocardial infarction (AMI)	1 hospitalization with an AMI diagnostic code	ICD-9: 410<br><br>ICD-10: I21	[8]
	Congestive heart failure (CHF)	1 hospitalization with a CHF diagnostic code<br><br>OR<br><br>2 physician visits in 1 year with CHF diagnostic code(s)	ICD-9: 428<br><br>ICD-10: I50	[8]
	Ischaemic heart disease (IHD)	Applicable to persons aged 20 years and older:<br><br>2 physician visits with Angina ICD-9 code 413 plus 1 prescription in 1 year<br><br>OR<br><br>1 specialist visit with Angina ICD-9 code 413 plus one prescription in 1 year<br><br>OR<br><br>2 physician visits with two ICD9 codes 410, 411, 412, 413, 414 in 1 year<br><br>OR	ICD-9: 410, 411, 412, 413, 414<br><br>ICD-10: I20, I21, I22, I23, I24, I25<br><br>Canadian Classification of Health Interventions (CCI): *CABG: 1IJ57LA, 1IJ57VS, 1IJ76 *PCI/PTCA: 1U50 ,1IJ57G<br><br>Canadian Classification of Diagnostic, Therapeutic, and Surgical Procedures (CCP): *CABG: 4811, 4812, 4813, 4814, 4815, 4816, 4817, 4819 *PCI/PTCA: 4802, 4803<br><br>*Note: CABG: coronary artery bypass surgery; PCI/PTCA: percutaneous coronary intervention/ Percutaneous transluminal coronary angioplasty	[8]

		1 CABG, PCI/PCTA procedure code<br><br>OR<br><br>1 hospitalization with any IHD code(s)		
	Cerebrovascular accident (stroke/CVA)	Applicable to persons aged 20 years and older:<br><br>1 hospitalization with a CVA diagnostic code<br><br>Note: Cases occurring on the same day as a traumatic brain injury event are excluded.	ICD-9: 362.3, 430, 431, 433.x1, 434, 435, 436<br><br>ICD-10: H34.1, I60, I61, I63, I64<br><br>Exclusions: any traumatic brain injury<br><br>ICD-9: 800, 801, 802, 803, 804, 850, 851, 852, 853, 854, V57.x<br><br>ICD-10: S02.x, S02.5, S06.x, Z50.x	[8]
	Transient Ischemic Attack (mini stroke/TIA)	Looking at persons aged 20 and older:<br><br>1 hospitalization with a TIA diagnostic code<br><br>Note: Cases occurring on the same day as a traumatic brain injury event are excluded. See exclusion codes for CVA above	ICD-9: 435<br><br>ICD-10: H34.0, G45.0, G45.1, G45.2, G45.3, G45.8, G45.9	[8]
Diabetes		1 hospitalization with a DM diagnostic code<br><br>OR	ICD-9: 250<br><br>ICD-10: E10, E11, E12, E13, E14	[8]

	2 physician visits in 1 year with DM diagnostic code(s)<br><br>OR<br><br>2 or more insulin prescriptions in 1 year<br><br>OR<br><br>2 or more oral antihyperglycemic (not including metformin) prescriptions in 1 year<br><br>OR<br><br>1 insulin and 1 oral antihyperglycemic (including metformin) in 1 year<br><br>OR<br><br>2 metformin prescriptions and 1 physician visit with diabetes code(s) in 1 year.<br><br>Note: Cases of suspected gestational diabetes in women aged 10-54 are not included by excluding hospitalizations, physician claims or prescriptions within the time period 120 days preceding or 180 days after hospital records containing	Drug Identification Numbers (DINs): 5894, 6009, 12556, 12564, 12599, 12602, 12610, 13730, 13889, 15598, 21350, 21849, 24708, 24716, 93033, 156663, 156728, 178543, 209872, 209937, 237000, 244449, 271330, 274119, 274127, 275409, 275417, 275425, 312711, 312762, 314552, 377937, 399302, 420336, 430986, 431168, 446564, 446572, 446580, 446599, 446602, 446610, 454753, 480290, 480304, 513644, 514535, 514551, 539201, 539244, 542911, 542938, 542946, 546348, 552259, 552267, 552275, 554820, 586714, 586773, 587737, 612162, 612170, 612189, 612197, 612200, 612219, 612227, 612235, 612243, 612251, 612278, 612359, 614416, 628301, 632651, 632678, 632686, 632694, 644358, 646148, 648094, 650935, 720933, 720941, 723789, 733075, 765996, 773654, 795879, 808733, 808741, 889091, 889105, 889113, 889121, 999717, 1900927, 1900935, 1913654, 1913662, 1913670, 1913689, 1934066, 1934074, 1934082, 1934090, 1934104, 1934112, 1959212, 1959220, 1959239, 1959352, 1959360, 1962639, 1962647, 1962655, 1962663, 1985930, 1985949, 1985957, 1985965, 1985973, 1985981, 1986085, 1986791, 1986805, 1986813, 1986821, 1987534, 1987542, 1987828, 1987836, 2020734, 2020742, 2022230, 2022249, 2024217, 2024225, 2024233, 2024241, 2024268, 2024276, 2024284, 2024292, 2024306, 2024314, 2024322, 2024403, 2024446, 2025248, 2025256, 2045710,	

	birth-related diagnostic codes (see gestational diabetes exclusion codes)	2084341, 2085887, 2099233, 2147521, 2147548, 2148765, 2155850, 2162822, 2162849, 2167786, 2188902, 2190885, 2190893, 2220628, 2223562, 2224550, 2224569, 2224771, 2224798, 2226804, 2226812, 2228920, 2228939, 2229516, 2229517, 2229519, 2229595, 2229596, 2229656, 2229704, 2229705, 2229785, 2229994, 2230026, 2230027, 2230036, 2230037, 2230443, 2230444, 2230475, 2230670, 2230671, 2231058, 2231095, 2231096, 2231389, 2233562, 2233999, 2234513, 2234514, 2236543, 2236548, 2236733, 2236734, 2236985, 2236986, 2237531, 2238103, 2238469, 2238470, 2238471, 2238698, 2238827, 2239081, 2239214, 2239474, 2239475, 2239476, 2239924, 2239925, 2239926, 2240294, 2240295, 2240297, 2241111, 2241112, 2241113, 2241114, 2241283, 2241310, 2242095, 2242096, 2242572, 2242573, 2242574, 2242589, 2242726, 2242783, 2242793, 2242794, 2242931, 2242974, 2242987, 2244353, 2245247, 2245272, 2245273, 2245274, 2245397, 2245438, 2245439, 2245440, 2245689, 2246820, 2246821, 2246964, 2246965, 2247085, 2247086, 2247087, 2248008, 2248009, 2248210, 2248440, 2248441, 2248453, 2251930, 2252945, 2252953, 2254719, 2257726, 2257734, 2258781, 2258803, 2258811, 2265435, 2265443, 2265575, 2265583, 2268493, 2268507, 2269031, 2269058, 2269589, 2269597, 2269600, 2269619, 2271842, 2273101, 2273128, 2273136, 2273756, 2273764, 2273772, 2274248, 2274256, 2274264, 2274272, 2274914, 2274922, 2274930, 2275864, 2275872,	

		2276410, 2279061, 2279088,<br>2279126, 2279460, 2279479,<br>2279487, 2284545, 2284553,<br>2284782, 2284790, 2287072,<br>2294338, 2294346, 2294400,<br>2295377, 2295385, 2295393,<br>2297795, 2297906, 2297914,<br>2297922, 2298279, 2298287,<br>2298295, 2300451, 2301423,<br>2301431, 2301458, 2302861,<br>2302888, 2302896, 2302942,<br>2302950, 2302977, 2303124,<br>2303132, 2303140, 2303442,<br>2303450, 2303469, 2303922,<br>2305062, 2306166, 2306174,<br>2306182, 2307170, 2307189,<br>2307197, 2307553, 2307561,<br>2307588, 2307634, 2307642,<br>2307650, 2307669, 2307677,<br>2307723, 2312050, 2312069,<br>2312077, 2313596, 2314894,<br>2314908, 2316544, 2320754,<br>2320762, 2320770, 2321475,<br>2321483, 2321491, 2326329,<br>2326337, 2326345, 2326477,<br>2326485, 2326493, 2331519,<br>2331527, 2333554, 2333856,<br>2333864, 2333872, 2334437,<br>2334445, 2336316, 2339110,<br>2339129, 2339587, 2339595,<br>2340763, 2340771, 2341522,<br>2341603, 2343606, 2343614,<br>2345366, 2345374, 2345382,<br>2345854, 2345862, 2348578,<br>2350459, 2350467, 2351056,<br>2351064, 2353377, 2353385,<br>2354144, 2354152, 2354160,<br>2354349, 2354357, 2354365,<br>2354926, 2354934, 2354942,<br>2355663, 2355671, 2355698,<br>2356422, 2357453, 2357461,<br>2357488, 2357887, 2357895,<br>2357909, 2357917, 2357925,<br>2361264, 2361272, 2361809,<br>2361817, 2363232, 2363240,<br>2363259, 2363518, 2363704,<br>2363712, 2364506, 2364514,<br>2365286, 2365294, 2365529,<br>2365537, 2366347, 2366355,<br>2366363, 2370921, 2373270,<br>2373289, 2373297, 2374013,	

		2374021, 2374048, 2374587, 2374595, 2375842, 2375850, 2375869, 2375877, 2377209, 2378043, 2378051, 2378116, 2378124, 2378620, 2378639, 2378841, 2378868, 2379767, 2379775, 2380196, 2380218, 2380722, 2380730, 2384906, 2384914, 2384922, 2385341, 2385368, 2388766, 2388774, 2388839, 2388847, 2389169, 2389177, 2389185, 2389290, 2389304, 2389312, 2391600, 2397307, 2403250, 2403269, 2403277, 2403366, 2403374, 2403382, 2403412, 2403420, 2403439, 2403447, 2405067, 2406020, 2406039, 2407124, 2408228, 2408236, 2409283, 2409291, 2412829, 2415089, 2415968, 2415976, 2415984, 2416786, 2416794, 2416808, 2417049, 2417057, 2417065, 2417189, 2417197, 2417200, 2417219, 2417227, 2417235, 2418002, 2418010, 2418029, 2419300, 2419319, 2419327, 2419335, 2419343, 2419351, 2421674, 2421682, 2421690, 2421828, 2421836, 2423286, 2424258, 2424266, 2424274, 2425483, 2425491, 2429764, 2429772, 2434121, 2434148, 2434156, 2435462, 2435470, 2437899, 2438275, 2438283, 2438658, 2439328, 2439611, 2441829, 2443635, 2443643, 2443937, 2443945, 2444844, 2444852, 2444933, 2444941, 2446065, 2448599, 2448602, 2448610, 2449390, 2449404, 2449765, 2449935, 2449943, 2455404, 2455412, 2455420, 2455439, 2455447, 2455455, 2456575, 2456583, 2456591, 2456605, 2456613, 2456621, 22303140, 66123203<br><br>Exclusions: gestational diabetes ICD-9: 641, 642, 643, 644, 645, 646, 647, 648,	

			650, 651, 652, 653, 654, 655, 656, 657, 658, 659, 660, 661, 662, 663, 664, 665, 666, 667, 668, 669, 670, 763, V27<br><br>ICD-10: O10, O11, O12, O13, O14, O15, O16, O21, O22, O23, O24, O25, O26, O28, O29, O30, O31, O32, O33, O34, O35, O36, O37, O40, O41, O42, O43, O44, O45, O46, O47, O48, O60, O61, O62, O63, O64, O65, O66, O67, O68, O69, O70, O71, O72, O73, O74, O75, O85, O86, O87, O88, O89, O90, O91, O92, O94, O95, O98, O99, Z37	
Hypertension (HTN)		Looking at persons aged 20 and older: 1 hospitalization with a hypertension diagnostic code<br><br>OR<br><br>2 physician visits in 2 years with hypertension diagnostic code(s)	ICD-9: 401, 402, 403, 404, 405<br><br>ICD-10: I10, I11, I12, I13, I15	[8]
Chronic kidney disease (CKD)	CKD	1 hospitalization with a CKD diagnostic code<br><br>OR<br><br>2 physician visits in 1 year with CKD diagnostic code(s)	ICD-9: 581, 582, 583, 585, 586, 587, 589<br><br>ICD-10: N01, N03, N04, N05, N06, N07, N18, N19, N26, N27	[8]
	Dialysis	9 or more physician visits within 90 days	Fee Codes: 00308, 00323, 00350, 00351, 00352, 00355, 00356, 00358, 00359, 00361,	

		with dialysis fee item code(s)	00390, 33708, 33723, 33750, 33751, 33752, 33755, 33756, 33758, 33759, 33761, 33790	
	Kidney transplant	1 hospitalization with procedure code(s) for kidney transplant	CCI: 675<br><br>CCP: 1PC85	[8]
History of Substance Use Disorder (SUD)		1 hospitalization with an opioid use disorder code<br><br>OR<br><br>3 or more physician visits with an opioid use disorder code<br><br>OR<br><br>1 or more opioid agonist treatment dispensation code<br><br>OR<br>A record indicating a history of injection drug use in the Drug Treatment Program registry	ICD-9: 39, 15039, 304.0, 304.7, 305.5, 965.0, E850.0-E850.2<br><br>ICD10: F11, X42 and (T40.0-T40.4 or T40.6), X62 and (T40.0-T40.4 or T40.6), Y12 and (T40.0-T40.4 or T40.6)<br><br>DINs or<br>Product Identification Numbers (PINs):<br>999792, 999793, 66999990, 66999991,6999992,66999993, 66999997, 66999998, 66999999, 67000000, 67000001,67000002, 67000003, 67000004, 67000005, 67000006, 67000007, 67000008, 2295695, 2295709, 2408090, 2408104, 2424851, 2424878, 2453908, 2453916, 2468085, 2468093, 2242962, 2242963, 2242964,66999994, 66999995, 66999996, 22123349, 2123346, 22123347, 2123348	[9]

Supplementary Material Figure S1. Flowchart describing study participant selection from the STOP HIV/AIDS PLWH cohort.

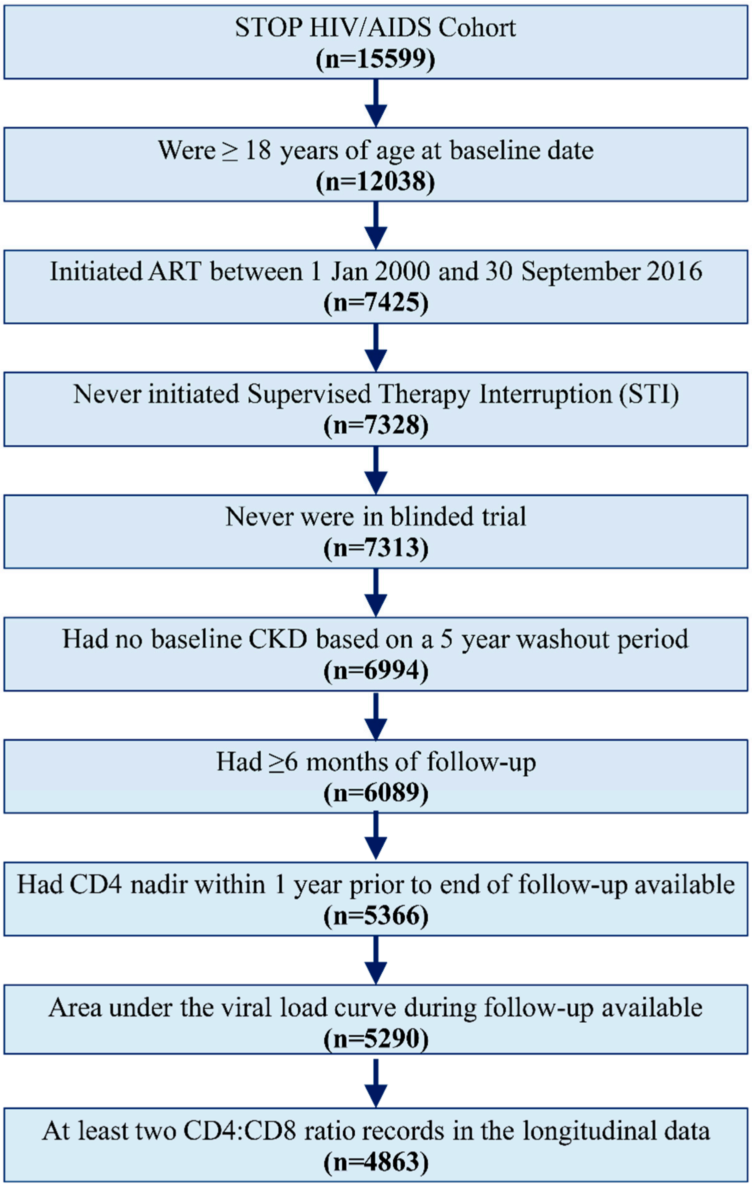

Note: CKD: Chronic Kidney Disease, ART: antiretroviral therapy.

Supplementary Material Table S2. Descriptive statistics of excluded and included participants.

	Excluded (N= 2450)	Included (N= 4863)	
**Risk factors**	**N (row %)**	**N (row %)**	**P-value**
**Sex**			
Female	497 (36.4)	867 (63.6)	
Male	1953 (32.8)	3996 (67.2)	0.0109
**SUD**			
No	1248 (31.0)	2784 (69.0)	
Yes	806 (32.4)	1685 (67.6)	
Unknown	396 (50.1)	394 (49.9)	<0.0001
**ART naïve**			
Yes	1875 (30.1)	4359 (69.9)	
No	575 (53.3)	504 (46.7)	<0.0001
**Year of baseline date**			
2000-2004	385 (27.3)	1026 (72.7)	
2005-2010	795 (28.3)	2012 (71.7)	
2011-2016	1270 (41.0)	1825 (59.0)	<0.0001
	**Median (Q1, Q3)**	**Median (Q1, Q3)**	
**Age at baseline (year)**	42 (35.0, 50.0)	41 (34.0, 49.0)	0.0219

Note: SUD: history of substance use disorder, ART: antiretroviral therapy.

Supplementary Material Table S3. Fit indices, entropy and model comparison of unconditional and including time-invariant covariates Growth Mixture Models.

Growth mixture models	AIC	BIC	SBIC	Adjusted LMR-LRT (P-value)	Entropy
Unconditional Models					
One-class (linear)	50083.37	50342.94	50215.84	---	---
Two-class (linear)	49258.81	49537.86	49401.22	0.007	0.947
Three-class (linear)	48891.62	49190.13	49043.96	0.030	0.954
One-class (quadratic)	43860.71	44146.24	44006.43	---	---
Two-class (quadratic)	42920.41	43231.91	43079.38	0.240	0.963
Model with covariates					
Two-class (linear) with time-invariant covariates	45538.22	46187.16	45869.40	<0.0001	0.945

Note: AIC: Akaike information criteria, BIC: Bayesian information criteria, SBIC: sample-size adjusted BIC, LMR-LRT: Adjusted Lo-Mendell-Rubin likelihood ratio test.

Supplementary Material Figure S2. Diagram of growth mixture model estimating growth parameters (i.e., intercept, slope) for linear trajectory shapes.

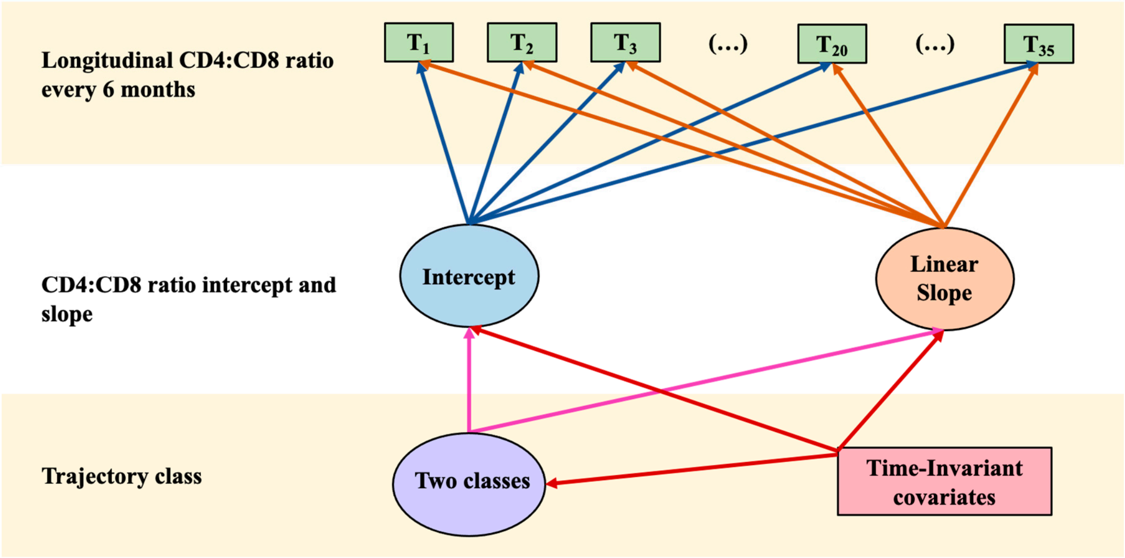

Supplementary Material Figure S3. Plots of the two-class (linear) models from the final Growth Mixture Models. A) Class 1 (N= 4547), B) Class 2 (N= 316).

A)

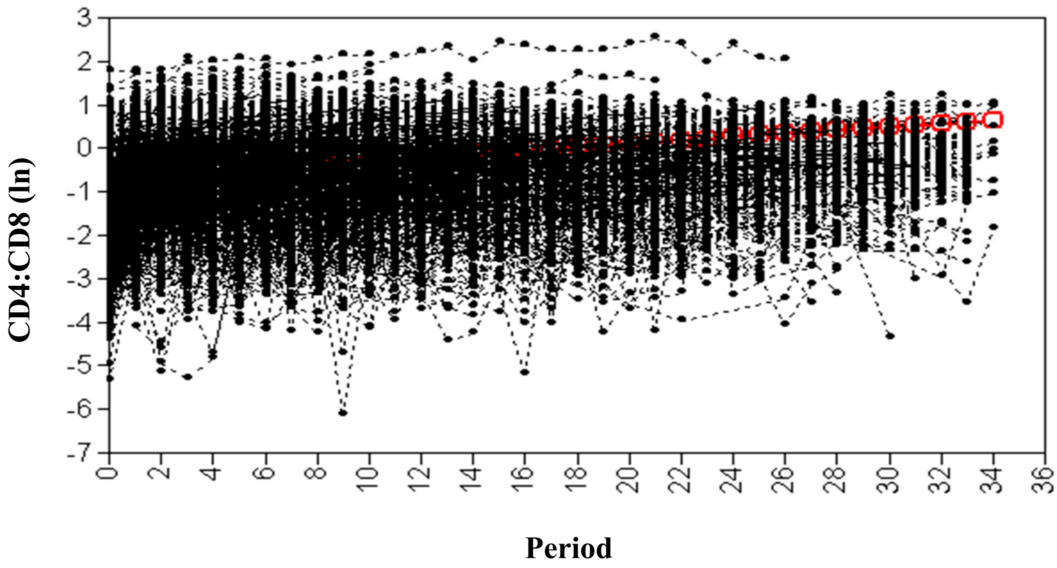

B)

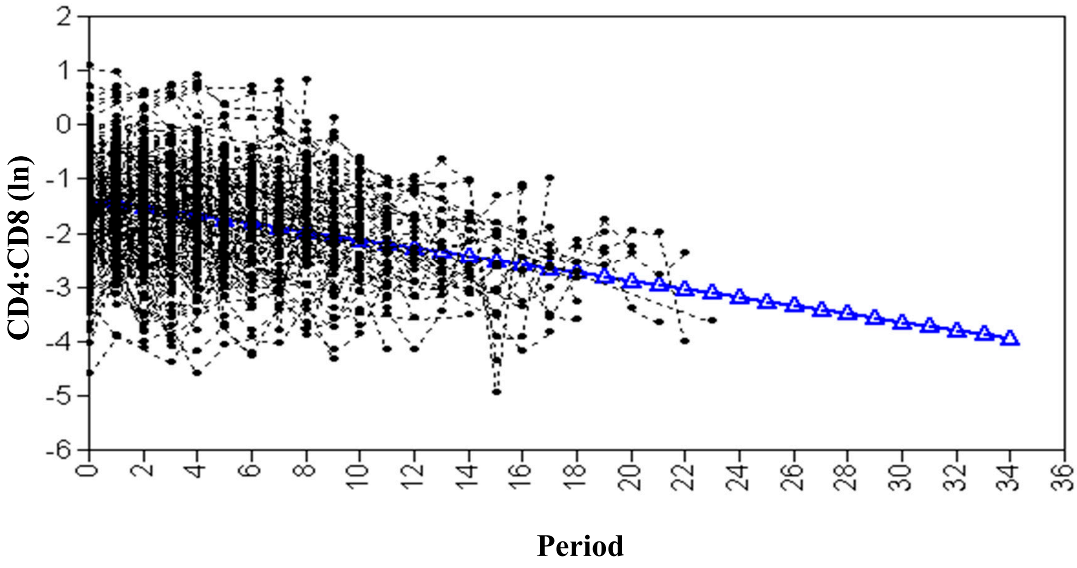

Decay model

Using the follow exponential decay model:

$$\ln CD4{:}CD8_{i,t} \sim \gamma e^{-R*t_i} + C_0 + b_{0i} + \varepsilon_{i,t}$$

where *t* represents each of the 6-month intervals; *i* represents each participant at the study, ε_i is the random error distributed as *Normal* (0, D_i), where *D* is the covariance matrix and b_{0i} as *N* (0, ϵ^2). The exponential decay function was modelled through γe^{-R*t}, where γ and R are coefficients of this function.

We obtained the parameters:

Supplementary Material Table S3. Goodness-of-fit parameters for the exponential decay model without covariates.

		**Class 1**		**Class 2**	
		**No CKD**	**CKD**	**No CKD**	**CKD**
**Parameters**	γ	-1.03	-0.99	-2.86	-0.29
	R	0.34	0.31	-0.03	-0.11
	C_0	-0.23	-0.49	1.56	-1.43

Supplementary Material Figure S4. CD4:CD8 Decay Model and Goodness-of-fit A) Class 1, no chronic kidney disease, B) Class 1, with chronic kidney disease, C) Class 2, no chronic kidney disease, D) Class 2, with chronic kidney disease

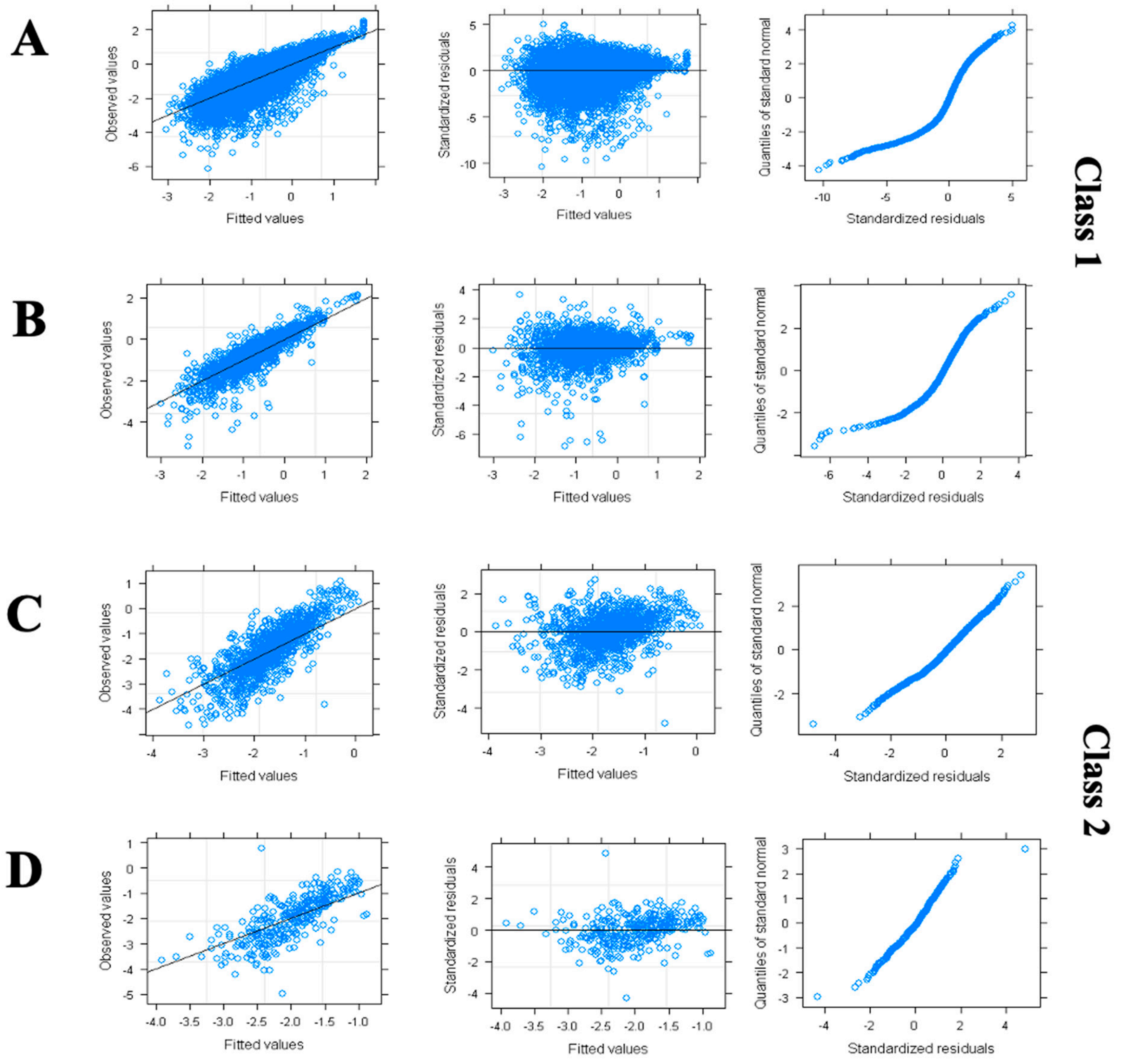

Supplementary Material Table S4. $\boldsymbol{ln(CD4:CD8)}$ trajectory and loss rate plots per six months by chronic kidney disease status among Class 2 participants.

	No CKD					CKD				
**Period (every 6 months)**	$\boldsymbol{r}$	$\boldsymbol{ln(r)}$	$\boldsymbol{\frac{dr}{dt}}$	$\boldsymbol{\frac{dy}{dt}}$	**% change**	$\boldsymbol{r}$	$\boldsymbol{ln(r)}$	$\boldsymbol{\frac{dr}{dt}}$	$\boldsymbol{\frac{dy}{dt}}$	**% change**
1	0.2530	-1.3743	-0.0187	-0.0741	-	0.1734	-1.7520	-0.0059	-0.0340	-
2	0.2347	-1.4494	-0.0178	-0.0760	7.2	0.1673	-1.7879	-0.0063	-0.0378	3.5
3	0.2173	-1.5263	-0.0169	-0.0779	7.4	0.1608	-1.8277	-0.0068	-0.0420	3.9
4	0.2009	-1.6052	-0.0160	-0.0799	7.6	0.1538	-1.8721	-0.0072	-0.0467	4.3
5	0.1852	-1.6861	-0.0152	-0.0819	7.8	0.1464	-1.9213	-0.0076	-0.0519	4.8
6	0.1705	-1.7691	-0.0143	-0.0840	8.0	0.1386	-1.9761	-0.0080	-0.0577	5.3
7	0.1566	-1.8542	-0.0135	-0.0862	8.2	0.1304	-2.0370	-0.0084	-0.0642	5.9
8	0.1435	-1.9415	-0.0127	-0.0884	8.4	0.1219	-2.1047	-0.0087	-0.0714	6.5
9	0.1312	-2.0310	-0.0119	-0.0906	8.6	0.1130	-2.1800	-0.0090	-0.0793	7.3
10	0.1197	-2.1228	-0.0111	-0.0930	8.8	0.1040	-2.2637	-0.0092	-0.0882	8.0
11	0.1089	-2.2169	-0.0104	-0.0953	9.0	0.0947	-2.3568	-0.0093	-0.0981	8.9
12	0.0989	-2.3135	-0.0097	-0.0978	9.2	0.0854	-2.4602	-0.0093	-0.1090	9.8
13	0.0896	-2.4125	-0.0090	-0.1003	9.4	0.0761	-2.5752	-0.0092	-0.1212	10.9
14	0.0809	-2.5141	-0.0083	-0.1028	9.7	0.0670	-2.7031	-0.0090	-0.1348	12.0
15	0.0729	-2.6182	-0.0077	-0.1055	9.9	0.0581	-2.8452	-0.0087	-0.1498	13.3
16	0.0655	-2.7251	-0.0071	-0.1082	10.1	0.0496	-3.0033	-0.0083	-0.1666	14.6
17	0.0587	-2.8346	-0.0065	-0.1109	10.4	0.0416	-3.1790	-0.0077	-0.1852	16.1
18	0.0525	-2.9469	-0.0060	-0.1138	10.6	0.0342	-3.3743	-0.0070	-0.2059	17.7
19	0.0468	-3.0622	-0.0055	-0.1167	10.9	0.0276	-3.5915	-0.0063	-0.2289	19.5
20	0.0416	-3.1803	-0.0050	-0.1197	11.1	0.0216	-3.8329	-0.0055	-0.2545	21.5
21	0.0368	-3.3015	-0.0045	-0.1227	11.4	0.0166	-4.1013	-0.0047	-0.2829	23.5
22	0.0325	-3.4258	-0.0041	-0.1259	11.7	0.0123	-4.3997	-0.0039	-0.3145	25.8
23	0.0286	-3.5533	-0.0037	-0.1291	12.0	0.0088	-4.7315	-0.0031	-0.3497	28.2

24	0.0251	-3.6840	-0.0033	-0.1324	12.3	0.0061	-5.1003	-0.0024	-0.3887	30.8
25	0.0220	-3.8181	-0.0030	-0.1358	12.5	0.0040	-5.5104	-0.0017	-0.4322	33.6
26	0.0191	-3.9555	-0.0027	-0.1392	12.8	0.0026	-5.9663	-0.0012	-0.4805	36.6
27	0.0166	-4.0966	-0.0024	-0.1428	13.2	0.0015	-6.4732	-0.0008	-0.5342	39.8
28	0.0144	-4.2412	-0.0021	-0.1464	13.5	0.0009	-7.0367	-0.0005	-0.5939	43.1
29	0.0124	-4.3895	-0.0019	-0.1502	13.8	0.0005	-7.6631	-0.0003	-0.6602	46.6
30	0.0107	-4.5416	-0.0016	-0.1540	14.1	0.0002	-8.3596	-0.0002	-0.7340	50.2
31	0.0091	-4.6976	-0.0014	-0.1580	14.4	0.0001	-9.1339	-0.0001	-0.8161	53.9
32	0.0078	-4.8575	-0.0013	-0.1620	14.8	4.56E-05	-9.9948	-4.14E-05	-0.9073	57.7
33	0.0066	-5.0216	-0.0011	-0.1661	15.1	1.75E-05	-10.9519	-1.77E-05	-1.0087	61.6
34	0.0056	-5.1899	-0.0009	-0.1704	15.5	6.05E-06	-12.0159	-6.78E-06	-1.1214	65.5
35	0.0047	-5.3624	-0.0008	-0.1748	15.8	1.85E-06	-13.1989	-2.31E-06	-1.2467	69.4

Note: CKD: chronic kidney disease, r: CD4:CD8 ratio, $ln(r)$: natural logarithm of CD4:CD8 ratio, $\frac{dr}{dt}$: increase or decrease rate of CD4:CD8 ratio, $\frac{dy}{dt}$: increase or decrease rate of natural logarithm of CD4:CD8 ratio.

Supplementary Material Table S5. $\boldsymbol{ln(CD4{:}CD8)}$ trajectory and loss rate plots per six months by chronic kidney disease status among Class 1 participants.

		No CKD					CKD				
**Period (every 6 months)**	**Year**	$\boldsymbol{r}$	$\boldsymbol{ln(r)}$	$\boldsymbol{\frac{dr}{dt}}$	$\boldsymbol{\frac{dy}{dt}}$	**% change**	$\boldsymbol{r}$	$\boldsymbol{ln(r)}$	$\boldsymbol{\frac{dr}{dt}}$	$\boldsymbol{\frac{dy}{dt}}$	**% change**
1	0.5	0.3842	-0.9565	0.2470	-	0.2988	-1.2078	0.2253		0.3842	-0.9565
2	1.0	0.4738	-0.7469	0.1763	23.3255	0.3627	-1.0142	0.1650	21.3624	0.4738	-0.7469
3	1.5	0.5503	-0.5973	0.1258	16.1368	0.4179	-0.8724	0.1208	15.2330	0.5503	-0.5973
4	2.0	0.6123	-0.4905	0.0897	11.2650	0.4637	-0.7686	0.0885	10.9415	0.6123	-0.4905
5	2.5	0.6608	-0.4144	0.0640	7.9142	0.5003	-0.6926	0.0648	7.9005	0.6608	-0.4144
6	3.0	0.6977	-0.3600	0.0457	5.5852	0.5289	-0.6369	0.0475	5.7265	0.6977	-0.3600
7	3.5	0.7252	-0.3212	0.0326	3.9541	0.5510	-0.5961	0.0348	4.1623	0.7252	-0.3212
8	4.0	0.7456	-0.2936	0.0233	2.8057	0.5677	-0.5662	0.0255	3.0314	0.7456	-0.2936
9	4.5	0.7605	-0.2738	0.0166	1.9940	0.5802	-0.5444	0.0186	2.2111	0.7605	-0.2738
10	5.0	0.7712	-0.2597	0.0118	1.4188	0.5896	-0.5283	0.0137	1.6145	0.7712	-0.2597
11	5.5	0.7790	-0.2497	0.0085	1.0103	0.5965	-0.5166	0.0100	1.1798	0.7790	-0.2497
12	6.0	0.7846	-0.2425	0.0060	0.7199	0.6017	-0.5080	0.0073	0.8626	0.7846	-0.2425
13	6.5	0.7887	-0.2374	0.0043	0.5131	0.6055	-0.5017	0.0054	0.6310	0.7887	-0.2374
14	7.0	0.7916	-0.2338	0.0031	0.3659	0.6083	-0.4971	0.0039	0.4617	0.7916	-0.2338
15	7.5	0.7936	-0.2311	0.0022	0.2609	0.6103	-0.4937	0.0029	0.3379	0.7936	-0.2311
16	8.0	0.7951	-0.2293	0.0016	0.1861	0.6118	-0.4913	0.0021	0.2473	0.7951	-0.2293
17	8.5	0.7962	-0.2280	0.0011	0.1328	0.6130	-0.4895	0.0015	0.1811	0.7962	-0.2280
18	9.0	0.7969	-0.2270	0.0008	0.0947	0.6138	-0.4881	0.0011	0.1326	0.7969	-0.2270
19	9.5	0.7974	-0.2263	0.0006	0.0676	0.6144	-0.4872	0.0008	0.0971	0.7974	-0.2263
20	10.0	0.7978	-0.2259	0.0004	0.0482	0.6148	-0.4865	0.0006	0.0711	0.7978	-0.2259
21	10.5	0.7981	-0.2255	0.0003	0.0344	0.6151	-0.4859	0.0004	0.0520	0.7981	-0.2255
22	11.0	0.7983	-0.2253	0.0002	0.0245	0.6154	-0.4856	0.0003	0.0381	0.7983	-0.2253
23	11.5	0.7984	-0.2251	0.0001	0.0175	0.6155	-0.4853	0.0002	0.0279	0.7984	-0.2251

24	12.0	0.7985	-0.2250	0.0001	0.0125	0.6156	-0.4851	0.0002	0.0204	0.7985	-0.2250
25	12.5	0.7986	-0.2249	0.0001	0.0089	0.6157	-0.4849	0.0001	0.0150	0.7986	-0.2249
26	13.0	0.7987	-0.2248	0.0001	0.0064	0.6158	-0.4848	0.0001	0.0110	0.7987	-0.2248
27	13.5	0.7987	-0.2248	3.82E-05	0.0045	0.6159	-0.4847	0.0001	0.0080	0.7987	-0.2248
28	14.0	0.7987	-0.2247	2.72E-05	0.0032	0.6159	-0.4847	0.0001	0.0059	0.7987	-0.2247
29	14.5	0.7987	-0.2247	1.94E-05	0.0023	0.6159	-0.4846	3.67E-05	0.0043	0.7987	-0.2247
30	15.0	0.7988	-0.2247	1.39E-05	0.0016	0.6159	-0.4846	2.69E-05	0.0032	0.7988	-0.2247
31	15.5	0.7988	-0.2247	9.89E-06	0.0012	0.6160	-0.4846	1.97E-05	0.0023	0.7988	-0.2247
32	16.0	0.7988	-0.2247	7.06E-06	0.0008	0.6160	-0.4846	1.44E-05	0.0017	0.7988	-0.2247
33	16.5	0.7988	-0.2247	5.04E-06	0.0006	0.6160	-0.4846	1.06E-05	0.0012	0.7988	-0.2247
34	17.0	0.7988	-0.2247	3.59E-06	0.0004	0.6160	-0.4845	7.73E-06	0.0009	0.7988	-0.2247
35	17.5	0.7988	-0.2247	2.56E-06	0.0003	0.6160	-0.4845	5.66E-06	0.0007	0.7988	-0.2247

Note: CKD: chronic kidney disease, r: CD4:CD8 ratio, $ln(r)$: natural logarithm of CD4:CD8 ratio, $\frac{dr}{dt}$: increase or decrease rate of CD4:CD8 ratio, $\frac{dy}{dt}$: increase or decrease rate of natural logarithm of CD4:CD8 ratio.

Scheme S6. $\boldsymbol{ln(CD4:CD8)}$ trajectory and loss rate plots per six months by chronic kidney disease status among Class 2 participants by sex.

		Class 2 No CKD							
		Male				Female			
**Period (every 6 months)**	**Year**	$\boldsymbol{r}$	$\boldsymbol{ln(r)}$	$\boldsymbol{\frac{dr}{dt}}$	$\boldsymbol{\frac{dy}{dt}}$	$\boldsymbol{r}$	$\boldsymbol{ln(r)}$	$\boldsymbol{\frac{dr}{dt}}$	$\boldsymbol{\frac{dy}{dt}}$
1	0.5	0.2467	-1.3994	-0.0177	-0.0718	0.2726	-1.2999	-0.0216	-0.0792
2	1.0	0.2294	-1.4723	-0.0170	-0.0740	0.2516	-1.3799	-0.0203	-0.0808
3	1.5	0.2128	-1.5473	-0.0162	-0.0761	0.2319	-1.4615	-0.0191	-0.0824
4	2.0	0.1970	-1.6246	-0.0154	-0.0784	0.2134	-1.5447	-0.0179	-0.0841
5	2.5	0.1819	-1.7041	-0.0147	-0.0807	0.1960	-1.6297	-0.0168	-0.0858
6	3.0	0.1676	-1.7860	-0.0139	-0.0831	0.1797	-1.7164	-0.0157	-0.0876
7	3.5	0.1541	-1.8703	-0.0132	-0.0855	0.1645	-1.8048	-0.0147	-0.0893
8	4.0	0.1413	-1.9571	-0.0124	-0.0880	0.1503	-1.8950	-0.0137	-0.0912
9	4.5	0.1292	-2.0464	-0.0117	-0.0906	0.1371	-1.9871	-0.0128	-0.0930
10	5.0	0.1178	-2.1384	-0.0110	-0.0933	0.1248	-2.0811	-0.0118	-0.0949
11	5.5	0.1072	-2.2331	-0.0103	-0.0961	0.1134	-2.1769	-0.0110	-0.0968
12	6.0	0.0972	-2.3306	-0.0096	-0.0989	0.1028	-2.2748	-0.0102	-0.0988
13	6.5	0.0880	-2.4309	-0.0090	-0.1018	0.0931	-2.3746	-0.0094	-0.1008
14	7.0	0.0793	-2.5342	-0.0083	-0.1048	0.0840	-2.4764	-0.0086	-0.1029
15	7.5	0.0713	-2.6406	-0.0077	-0.1079	0.0758	-2.5803	-0.0080	-0.1050
16	8.0	0.0639	-2.7501	-0.0071	-0.1111	0.0681	-2.6863	-0.0073	-0.1071
17	8.5	0.0571	-2.8628	-0.0065	-0.1144	0.0611	-2.7945	-0.0067	-0.1093
18	9.0	0.0509	-2.9788	-0.0060	-0.1177	0.0548	-2.9049	-0.0061	-0.1115
19	9.5	0.0451	-3.0983	-0.0055	-0.1212	0.0489	-3.0175	-0.0056	-0.1138
20	10.0	0.0399	-3.2212	-0.0050	-0.1248	0.0436	-3.1324	-0.0051	-0.1161
21	10.5	0.0352	-3.3479	-0.0045	-0.1285	0.0388	-3.2497	-0.0046	-0.1184
22	11.0	0.0309	-3.4782	-0.0041	-0.1322	0.0344	-3.3693	-0.0042	-0.1208

23	11.5	0.0270	-3.6124	-0.0037	-0.1361	0.0305	-3.4914	-0.0038	-0.1233
24	12.0	0.0235	-3.7505	-0.0033	-0.1401	0.0269	-3.6159	-0.0034	-0.1258
25	12.5	0.0204	-3.8927	-0.0029	-0.1443	0.0237	-3.7430	-0.0030	-0.1284
26	13.0	0.0176	-4.0391	-0.0026	-0.1485	0.0208	-3.8727	-0.0027	-0.1310
27	13.5	0.0151	-4.1898	-0.0023	-0.1529	0.0182	-4.0050	-0.0024	-0.1336
28	14.0	0.0130	-4.3450	-0.0020	-0.1574	0.0159	-4.1400	-0.0022	-0.1364
29	14.5	0.0111	-4.5047	-0.0018	-0.1621	0.0139	-4.2778	-0.0019	-0.1391
30	15.0	0.0094	-4.6691	-0.0016	-0.1668	0.0121	-4.4183	-0.0017	-0.1420
31	15.5	0.0079	-4.8384	-0.0014	-0.1718	0.0104	-4.5617	-0.0015	-0.1449
32	16.0	0.0067	-5.0127	-0.0012	-0.1768	0.0090	-4.7081	-0.0013	-0.1478
33	16.5	0.0056	-5.1921	-0.0010	-0.1820	0.0078	-4.8574	-0.0012	-0.1508
34	17.0	0.0046	-5.3768	-0.0009	-0.1874	0.0067	-5.0097	-0.0010	-0.1539
35	17.5	0.0038	-5.5670	-0.0007	-0.1929	0.0057	-5.1651	-0.0009	-0.1570

Class 2 CKD

		**Male**				**Female**			
**Period (every 6 months)**	**Year**	$\boldsymbol{r}$	$\boldsymbol{ln(r)}$	$\boldsymbol{\frac{dr}{dt}}$	$\boldsymbol{\frac{dy}{dt}}$	$\boldsymbol{r}$	$\boldsymbol{ln(r)}$	$\boldsymbol{\frac{dr}{dt}}$	$\boldsymbol{\frac{dy}{dt}}$
1	0.5	0.1661	-1.7953	-0.0050	-0.0299	0.1882	-1.6703	-0.0073	-0.0387
2	1.0	0.1609	-1.8270	-0.0054	-0.0335	0.1806	-1.7113	-0.0079	-0.0436
3	1.5	0.1553	-1.8625	-0.0058	-0.0375	0.1725	-1.7576	-0.0085	-0.0491
4	2.0	0.1492	-1.9022	-0.0063	-0.0420	0.1637	-1.8097	-0.0091	-0.0553
5	2.5	0.1428	-1.9466	-0.0067	-0.0470	0.1544	-1.8685	-0.0096	-0.0623
6	3.0	0.1358	-1.9964	-0.0071	-0.0526	0.1445	-1.9347	-0.0101	-0.0702
7	3.5	0.1285	-2.0520	-0.0076	-0.0589	0.1341	-2.0092	-0.0106	-0.0791
8	4.0	0.1207	-2.1144	-0.0080	-0.0659	0.1233	-2.0933	-0.0110	-0.0892
9	4.5	0.1126	-2.1841	-0.0083	-0.0738	0.1121	-2.1880	-0.0113	-0.1005
10	5.0	0.1041	-2.2622	-0.0086	-0.0825	0.1008	-2.2947	-0.0114	-0.1132

11	5.5	0.0954	-2.3495	-0.0088	-0.0924	0.0894	-2.4150	-0.0114	-0.1276
12	6.0	0.0865	-2.4473	-0.0089	-0.1034	0.0780	-2.5505	-0.0112	-0.1438
13	6.5	0.0776	-2.5568	-0.0090	-0.1157	0.0670	-2.7032	-0.0109	-0.1620
14	7.0	0.0686	-2.6793	-0.0089	-0.1295	0.0564	-2.8752	-0.0103	-0.1825
15	7.5	0.0598	-2.8164	-0.0087	-0.1450	0.0465	-3.0691	-0.0096	-0.2057
16	8.0	0.0513	-2.9699	-0.0083	-0.1623	0.0373	-3.2875	-0.0087	-0.2318
17	8.5	0.0432	-3.1417	-0.0078	-0.1816	0.0292	-3.5337	-0.0076	-0.2612
18	9.0	0.0357	-3.3340	-0.0072	-0.2033	0.0221	-3.8111	-0.0065	-0.2943
19	9.5	0.0287	-3.5492	-0.0065	-0.2275	0.0162	-4.1236	-0.0054	-0.3316
20	10.0	0.0226	-3.7900	-0.0058	-0.2547	0.0114	-4.4759	-0.0043	-0.3736
21	10.5	0.0173	-4.0596	-0.0049	-0.2851	0.0077	-4.8727	-0.0032	-0.4210
22	11.0	0.0128	-4.3614	-0.0041	-0.3191	0.0049	-5.3199	-0.0023	-0.4744
23	11.5	0.0091	-4.6991	-0.0033	-0.3571	0.0030	-5.8238	-0.0016	-0.5346
24	12.0	0.0062	-5.0771	-0.0025	-0.3997	0.0017	-6.3917	-0.0010	-0.6024
25	12.5	0.0041	-5.5002	-0.0018	-0.4474	0.0009	-7.0315	-0.0006	-0.6788
26	13.0	0.0025	-5.9737	-0.0013	-0.5007	0.0004	-7.7525	-0.0003	-0.7649
27	13.5	0.0015	-6.5037	-0.0008	-0.5604	0.0002	-8.5649	-0.0002	-0.8619
28	14.0	0.0008	-7.0970	-0.0005	-0.6273	0.0001	-9.4804	-0.0001	-0.9712
29	14.5	0.0004	-7.7609	-0.0003	-0.7021	0.0000	-10.5120	0.0000	-1.0944
30	15.0	0.0002	-8.5041	-0.0002	-0.7858	0.0000	-11.6744	0.0000	-1.2332
31	15.5	0.0001	-9.3359	-0.0001	-0.8795	0.0000	-12.9842	0.0000	-1.3896
32	16.0	0.0000	-10.2669	0.0000	-0.9844	0.0000	-14.4601	0.0000	-1.5658
33	16.5	0.0000	-11.3089	0.0000	-1.1018	0.0000	-16.1233	0.0000	-1.7644
34	17.0	0.0000	-12.4752	0.0000	-1.2332	0.0000	-17.9973	0.0000	-1.9882
35	17.5	0.0000	-13.7806	0.0000	-1.3803	0.0000	-20.1090	0.0000	-2.2403

Note: CKD: chronic kidney disease, r: CD4:CD8 ratio, $ln(r)$: natural logarithm of CD4:CD8 ratio, $\frac{dr}{dt}$: increase or decrease rate of CD4:CD8 ratio, $\frac{dy}{dt}$: increase or decrease rate of natural logarithm of CD4:CD8 ratio.

References cited in the Supplemental Material

1. British Columbia Centre for Disease Control. *HIV/AIDS Information System (HAISYS)*; British Columbia Centre for Disease Control: Vancouver, BC, Canada. Available online: http://www.bccdc.ca/about/accountability/data-access-requests/public-health-data (accessed on 12 October 2021).
2. British Columbia Centre for Disease Control Public Health Laboratory. *HIV Laboratory Testing Datasets (Tests: ELISA, Western blot, NAAT, p24, Culture)*; British Columbia Centre for Disease Control Public Health Laboratory: Vancouver, BC, Canada.
3. British Columbia Centre for Excellence in HIV/AIDS (BCCfE). Drug Treatment Program. Available online: http://cfenet.ubc.ca/drug-treatment-program (accessed on 12 October 2021).
4. Patterson, S.; Cescon, A.; Samji, H.; Cui, Z.; Yip, B.; Lepik, K.J.; Moore, D.; Lima, V.D.; Nosyk, B.; Harrigan, P.R.; et al. Cohort Profile: HAART Observational Medical Evaluation and Research (HOMER) cohort. *Int J Epidemiol* **2015**, *44*, 58-67, doi:10.1093/ije/dyu046.
5. British Columbia Ministry of Health [**creator**] (2016):. Medical Services Plan (MSP) Payment Information File; Consolidation File (MSP Registration & Premium Billing); Home & Community Care (Continuing Care); Mental Health; PharmaNet. . Available online: http://www2.gov.bc.ca/gov/content/health/conducting-health-research-evaluation/data-access-health-data-central. (accessed on 12 October 2021).
6. Canadian Institute for Health Information. *Discharge Abstract Database (Hospital Separations)*; Canadian Institute for Health Information: Ottawa, ON, Canada, 2016.
7. British Columbia Vital Statistics Agency. *Vital Statistics*; British Columbia Vital Statistics Agency: Vancouver, BC, Canada, 2016.
8. Chronic Disease Information Working Group. BC Chronic Disease and Selected Procedure Case Definitions version 2015. Available online: https://www2.gov.bc.ca/assets/gov/health/conducting-health-research/data-access/chronic-disease-registries-case-definitions.pdf (accessed on 12 October 2021).
9. Piske, M.; Zhou, H.; Min, J.E.; Hongdilokkul, N.; Pearce, L.A.; Homayra, F.; Socias, M.E.; McGowan, G.; Nosyk, B. The cascade of care for opioid use disorder: a retrospective study in British Columbia, Canada. *Addiction* **2020**, *115*, 1482-1493, doi:10.1111/add.14947.
